# The Submental Nasal Appearance Scale for the Assessment of Repaired Unilateral Complete Cleft Lip: A Validation Study

**DOI:** 10.1177/1055665620961968

**Published:** 2020-10-06

**Authors:** R. A. Tan, I. E. Schipper, H. C. W. de Vet, J. P. W. Don Griot

**Affiliations:** 1 Department of Plastic, Reconstructive and Hand Surgery, 1209 Amsterdam UMC, location VUMC, Amsterdam, the Netherlands; 2 Department of Epidemiology and Biostatistics and the Amsterdam Public Health Research Institute, 1209 Amsterdam UMC, location VUmc, Amsterdam, the Netherlands

**Keywords:** cleft lip and palate, basal view, nasal appearance, assessment method

## Abstract

**Objective:**

To reassess reliability and validity of the Submental Nasal Appearance Scale (SNAS) compared to the preliminary pilot study, for assessment of patient photographs with repaired unilateral cleft lip and palate (UCLP). When utilizing the SNAS, 3 nasal features (1. nasal outline; 2. alar base position; 3. nostril axis) must be graded according to symmetry between the cleft and noncleft side using a 5-point scale with reference photographs for each feature. The mean score calculated from the graded features reflects the overall degree of nasal symmetry, which is considered an important goal when repairing UCLP.

**Design:**

Fifty patient photographs were selected and cropped, displaying the submental view. Six raters assessed these photographs using the SNAS and a separate 5-point scale to assess the overall submental appearance. Interrater reliability was determined for both methods and correlation was calculated between these as an indication of construct validity.

**Setting:**

Amsterdam UMC, location VUmc, Amsterdam, The Netherlands.

**Patients:**

Six- to 9-year-old patients with repaired UCLP.

**Results:**

Interrater reliability of 0.73 and 0.48 was found for the SNAS and overall appearance assessment, respectively, while in the pilot study values of 0.79 and 0.69 were found. Correlation of 0.59 and 0.74 was found in the current and pilot study, respectively, between the SNAS and overall appearance assessment.

**Conclusions:**

The SNAS is a reliable tool to assess nasal symmetry from the submental perspective. Reliability of the SNAS is higher compared to grading overall appearance, but validity of the SNAS was less well supported.

## Introduction

In patients with unilateral cleft lip and palate (UCLP), nasolabial appearance can be denoted as a highly important factor with major impact on patient’s perceived quality of life (Mani et al., 2013; Wong Riff et al., 2018; Zeraatkar et al., 2019). In order to document and compare such appearance outcomes, a reliable and internationally accepted evaluation method is still required (Al-Omari et al., 2005; Sharma et al., 2012; Mosmuller et al., 2013).

Hence, Tan et al. (2019) conducted a pilot study to assess nasal appearance on submental view photographs of 6- to 9-year-old patients with repaired UCLP. In the pilot study, the usefulness of 2-dimensional (2D) photographs and the advantages of grading from the submental perspective were emphasized. Important nasal structures are clearly visualized from this perspective, making it convenient to assess symmetry, which is a crucial element a surgeon strives to achieve when repairing UCLP (Baudouin and Tiberghien, 2004; Mulliken and LaBrie, 2012; He et al., 2015; Knight et al., 2016).

**Figure 1.**
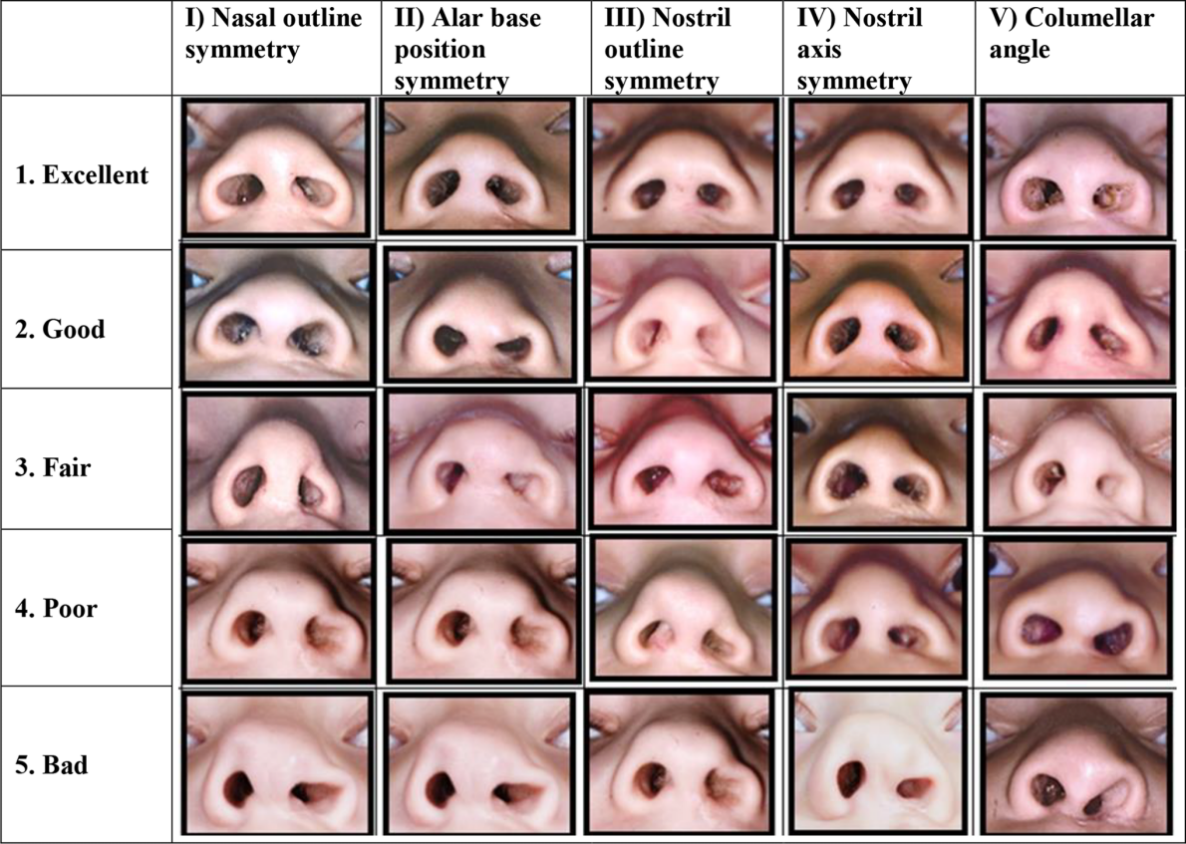
Sets of reference photographs for 5 submental characteristics.

**Figure 2.**
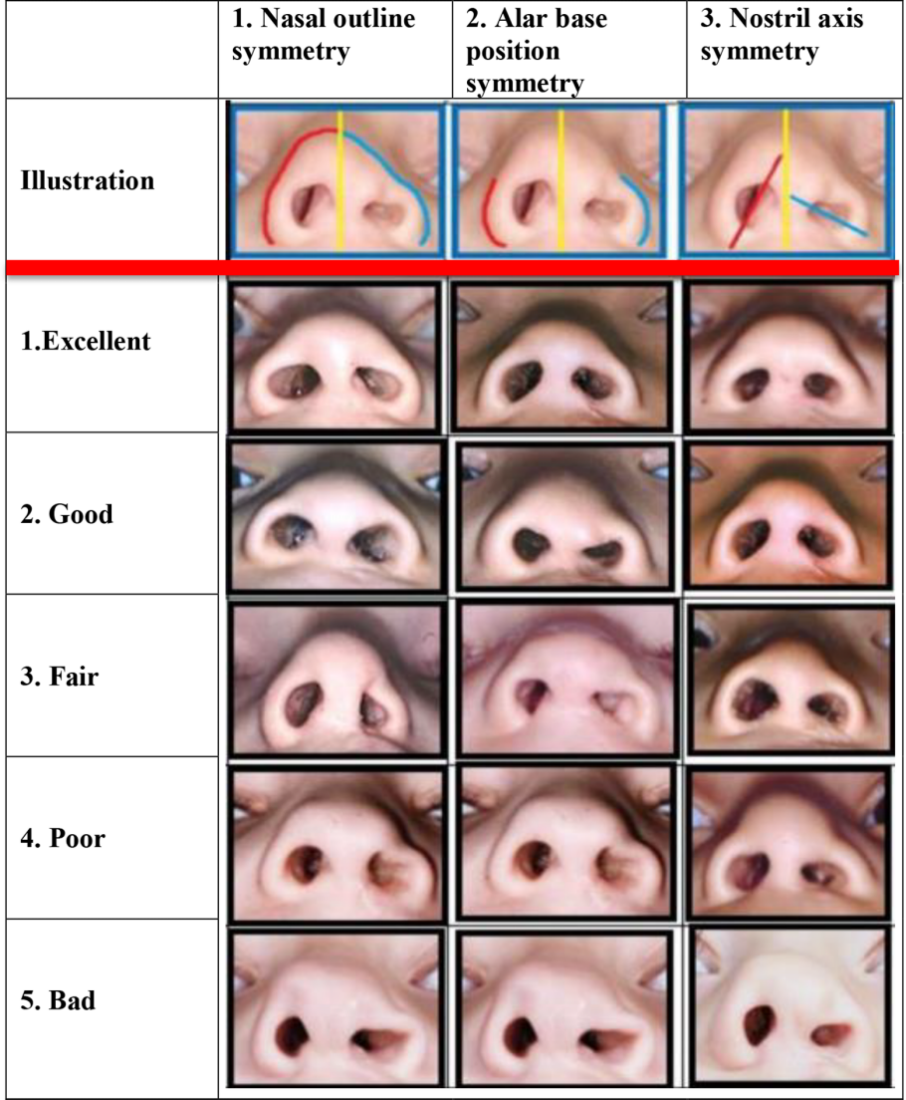
The Submental Nasal Appearance Scale (SNAS).

Tan et al. (2019) created a set of 5 reference photographs to represent 5 degrees (1 = excellent; 2 = good; 3 = fair; 4 = poor; and 5 = bad) of symmetry between the cleft and the noncleft side for each of the following 5 important nasal structures: (1) nasal outline; (2) alar base position; (3) nostril outline; (4) nostril axis; and (5) columellar angle (Figure 1). The mean degree score of combination I + II + IV predicted the highest intrarater reliability (0.84) and the second highest interrater reliability (0.79) when 3 cleft surgeons assessed 24 nasal photographs twice using the reference photographs. In order to test the construct validity of these symmetry scores, correlation with assessment of overall submental appearance (ie, surgeon’s/expert’s opinion on gestalt) was determined, as this could be considered as a gold standard in assessment of nasolabial appearance. It appeared that combination I + II + IV yielded the highest correlation (0.74) compared to overall nasal appearance from the submental perspective. As a result, it was decided to simplify the 5 characteristics to 3 using the latter combination, which resulted in the Submental Nasal Appearance Scale (SNAS; Figure 2).

The strong correlation obtained in the pilot study suggested that the degree of symmetry obtained with the SNAS could resemble the degree of overall submental appearance, though in a more reliable way (Tan et al., 2019).

Before the SNAS can be used in daily practice, reliability and validity needs to be reassessed. The specific aims were to (1) determine interrater reliability for grading symmetry with the SNAS, using a set of reference photographs for the 3 characteristics instead of 5. To (2) determine interrater reliability for assessment of the overall submental appearance and to (3) determine correlation between grading symmetry with the SNAS and the assessment of overall submental appearance.

## Method and Materials

Photographs of patients used in this reliability and validation study have been drawn from the Academic Center for Dentistry Amsterdam (ACTA) database, as these patients received treatment at Amsterdam UMC, location VUmc and the ACTA both in The Netherlands.

Fifty submental view photographs of 6- to 9-year-old patients with repaired UCLP treated over the past 30 years were obtained. These photographs were randomly selected to ensure that the spectrum of nasal symmetry was representative for patients seen by cleft surgeons. The photographs had to meet similar selection criteria as described in the pilot study: Patients displayed must have had a neutral facial expression, and cases with former facial trauma affecting the nasolabial region were excluded. Photographs were manually set in the horizontal plane.

**Figure 3.**
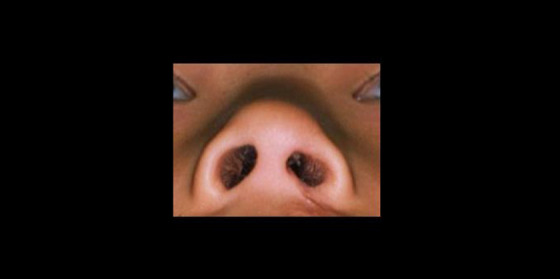
Slide containing a live-sized nose. Both canthi are displayed.

Next, the photographs were cropped and presented as being left-sided clefts, displaying the nose from the submental view including both canthi and were placed on a Microsoft PowerPoint (Microsoft Corp) slide, with size approaching a live-sized nose (Figure 3). Cropping was done in order to reduce the influence of related facial structures, such as eyes and ears, which potentially could influence a rating task (Prahl et al., 2006; Bongaarts et al., 2008). Image quality, lighting differential, and skin color were not uniformly adjusted in the current study, contrary to the pilot study.

The 50 PowerPoint slides containing the photographs were randomly divided into 2 series, resulting in 2 series containing 25 photographs. Both series were separately assessed on 2 different occasions by 6 raters, consisting of 2 plastic surgeons (P1 and P2), 2 maxillofacial surgeons (M1 and M2), and 2 orthodontists (O1 and O2). All raters were involved in cleft lip and palate treatment. One of the authors (I.E.S.) visited all raters in their treatment office, where raters separately performed the assessment task, behind a desktop/laptop and results were written down on a score form. A time limit for the assessment task was not utilized. Dividing the number of 50 photographs into halves was done in order to avoid loss of concentration among the raters.

In between each slide containing a photograph, a blank slide was incorporated. During the rating task, this slide was shown for 10 seconds in between grading the nasal photographs, in order to reduce memory bias arising from grading the previous nose.

Prior to the assessment of both series, the raters received a brief instruction and graded 3 practice photographs to become familiar with the SNAS. To each of the nasal photographs, 3 feature (1. nasal outline + 2. alar base position + 3. Nostril axis) scores were given according to symmetry using the SNAS (3 sets of reference photographs) and immediately after the overall submental appearance was scored (before grading the subsequent nose) using a 5-point scale (1 = very good; 2 = good; 3 = moderate; 4 = poor; and 5 = bad) without reference photographs. The scores of the 3 symmetry features were averaged to obtain the SNAS score (rounded up to the first decimal), and this score was used for the statistical analyses. Interrater reliability was determined for these SNAS scores. In addition, interrater reliability was determined for the overall appearance scale. Correlation between the obtained SNAS scores and the overall appearance scores was determined as an indication for construct validity. To estimate a time indication for assessment of a single photograph, the mean duration for each rater was recorded. The sum of the duration of 6 raters (first occasion) was calculated after which the practice time and 10-second intervals were subtracted. The total number of photographs assessed eventually divided this number, which resulted in the mean assessment time per photograph.

### Statistical Analysis

The statistical program IBM SPSS (IBM Corp) 24.0 was used for all data analysis. The intraclass correlation coefficient (ICC; 2-way random model with absolute agreement) was used to determine the interrater reliability of the SNAS scores and the scores obtained when grading the overall appearance. An upper limit of 1.0 can be used concerning the ICC, which indicates a high level of agreement. An ICC score of 0.70 is considered to be the minimal acceptable value for research purposes, and a score of 0.90 or higher is acceptable for clinical use (De Vet et al., 2011). To strive after a minimum ICC of 0.70, a sample size of 50 photographs was required (De Vet et al., 2011).

The correlation between the obtained SNAS scores and the overall appearance scores was determined by using the Kendall’s Tau rank correlation coefficient. The following classification for correlation can be utilized: 0-0.30 = negligible; 0.30-0.50 = poor; 0.50-0.70 = moderate; 0.70-0.90 = high; and above 0.90 = very high (Hinkle et al., 2003).

## Results

The 50 photographs used consisted of 38 (76%) patients with repaired left-sided clefts and 33 (66%) were female. The mean duration to assess a single photograph with the SNAS and the overall appearance scale was 13.4 seconds. The following distribution of scores (degree 1-5) among the 6 raters was obtained for each of the 3 components: I. nasal outline (1 = 6.7%; 2 = 28%; 3 = 40%; 4 = 21.3%; and 5 = 4%), II. alar base position (1 = 5.3%; 2 = 28%; 3 = 40%; 4 = 23.4%; and 5 = 3.3%), and III. nostril axis (1 = 0.0%; 2 = 14.7%; 3 = 34%; 4 = 35.3%; and 5 = 16%).

Table 1 illustrates the interrater reliability between the 6 raters obtained with the SNAS and the scale to grade overall appearance. The interrater reliability obtained with the SNAS was 0.73. For grading the overall appearance, an interrater reliability of 0.48 was found.

**Table 1.** Interrater Reliability of the Submental Nasal Appearance Scale (SNAS) and the Overall Submental Appearance Scale.

Assessment method	ICC	Spearman-Brown
SNAS	0.734	0.892
Overall appearance	0.484	0.738

Abbreviations: ICC, intraclass correlation coefficient; SNAS, Submental Nasal Appearance Scale.

The Kendall’s Tau correlation between the SNAS and the overall appearance scale is illustrated in Table 2. The 6 raters obtained a mean correlation of 0.59. Raters P1 and P2 obtained values of 0.31 and 0.49. Raters M1 and M2 both obtained correlation values of 0.72, while rater O1 obtained a value of 0.78 and rater O2 obtained a value of 0.59.

**Table 2.** Kendall’s Tau Correlation Between the SNAS and the Overall Submental Appearance Scale.

Rater	Kendall’s Tau correlation coefficient
P1	0.308
P2	0.485
M1	0.715
M2	0.718
O1	0.779
O2	0.541
Mean	0.591

Abbreviation: SNAS, Submental Nasal Appearance Scale.

## Discussion

The goal of the current study was to test the SNAS on its reliability and validity in order to confirm the results of the pilot study, before it might be used for research and/or clinical purposes on 6- to 9-year-old patients with repaired UCLP. Hence, 3 specific aims were outlined in the introduction section: to (1) determine interrater reliability for grading symmetry with the SNAS, using the 3 sets of reference photographs instead of 5. To (2) determine interrater reliability for assessment of overall submental appearance and to (3) determine correlation between grading symmetry with the SNAS and the assessment of overall appearance. To reach the 3 aims, 6 raters performed the assessments.

The SNAS exhibited an interrater reliability of 0.73 in this study, while in the pilot study an interrater reliability of 0.79 was obtained. Although the interrater reliability value obtained in the current study is slightly lower, it can be assumed that the SNAS is reliable enough to use for research purposes (minimum acceptable value of 0.70) when a single rater uses it in future studies. The Spearman-Brown value (Table 1) illustrates an estimated interrater reliability for 3 raters performing assessment with the SNAS, resulting in a reliability increasing toward a value of 0.89, which nearly approaches the value of 0.90, which can be considered as the minimum acceptable value for an instrument to be appropriate for clinical purposes, according to De Vet et al. (2011).

Comparing the interrater reliability of the SNAS with the overall appearance scale it appears in both the current and the pilot study, that the SNAS can be utilized in a substantially more reliable way since values of 0.73 (SNAS) and 0.48 (overall appearance) were found in the current study compared to values of 0.79 (SNAS) and 0.62 (overall appearance) in the pilot study. Even if the overall appearance scores of 3 raters were averaged according to the Spearman-Brown, the estimated value of 0.74 for overall appearance did not approach the value of 0.90 for use in clinical practice.

The third aim of this study was to test the validity of the SNAS compared to the overall nasal appearance scale. A substantially lower mean Kendall’s Tau rank correlation of 0.59 was found in the current study compared to the value of 0.74 found in the pilot study. This implies that grading symmetry versus the overall submental appearance (ie, aesthetics) is not in complete concordance. For this reason, construct validity of the SNAS to assess overall nasal appearance is not supported in the current study.

The SNAS is considered a subjective assessment instrument since the scores given to each of the 3 features is depending on the subjective opinion of the rater. Despite the use of reference photographs that makes the rating task appear to be easier, the quality of symmetry assessment is depending on the ability of the rater to distinguish between the various degrees of symmetry between the cleft and noncleft side. In our opinion, this is imperatively related to the rater’s experience with UCLP treatment and whether raters are trained in utilizing the SNAS or SNAS-like assessment methods. All raters in the current study were experienced in treatment of UCLP, but were newly introduced to the SNAS when performing the assessments. This forms the main explanation for the variation found between interrater reliability values (0.73 and 0.79) of the current and pilot study. The interrater reliability found in the current study, however, is still higher than the preliminary submental rating methods described. He et al. (2009) reported a comparable reliability of 0.72; however, this value was obtained combining the submental, frontal, and lateral view. The exhibited reliability for the submental view solely was 0.64. Rubin et al. (2015) described a submental method to facilitate the frontal/lateral assessment method of Asher-McDade et al. (1991). They selected reference photographs to address different degrees of nasal form and nasal symmetry but also found a lower interrater reliability of 0.68 for their method. Moreover, the method of Rubin et al. (2015) is not yet validated. Both studies were already extensively discussed in the pilot study (Tan et al., 2019).

Subjectivity plays even a larger role in assessment of the overall appearance. This is expressed by the remarkably lower interrater reliability values of 0.48 and 0.62 obtained in both the current and the pilot study, respectively. This subjectivity also affects the correlation between the SNAS and the overall appearance scale, as in the current study, a value of 0.59 was found and in the pilot study, a value of 0.74 was obtained. First of all, this correlation discrepancy between the current and the pilot study can be explained by the fact that, concerning the pilot study, 3 cleft surgeons, who worked within the same cleft unit, highly focused on symmetry during assessment of overall appearance, while the 6 raters of different disciplines in the current study could have had more focus on specific nasal aesthetic morphology. Secondly, 2 of the 3 cleft surgeons that performed assessment in the pilot study were involved in development of the sets of reference photographs regarding the 5 characteristics. This meant that they already had assessed 61 submental view noses before performing the assessment on the 24 submental photographs to determine the reliability and correlation. This might have led to higher reliability values in the pilot study.

In the current study, rater P1, P2, and O2 obtained remarkably lower values compared to the rest of the raters. Some possible explanations to address these differences occurred after analyzing some of the photographs that received scores that correlated poorly. Raters P1 and P2 specifically mentioned that they intended to grade a nose with a broad nasal tip as poorer, though this could have been a symmetrical outcome according to the SNAS. In addition, the raters in this study mentioned that the nasal tip outline of the reference photograph (Figure 2) that represents the “fair” outcome for “nasal outline symmetry” was relatively small compared to the other reference photographs representing the nasal outline symmetry. Although the raters were instructed to only assess symmetry, this could have made some of the raters inclined to score the smaller noses as fair, which can also clarify the differences in correlation with appearance scale.

As it appeared that photographs could be assessed in a more reliable way using the SNAS compared to overall appearance assessment and moreover high correlation between the SNAS and overall appearance was found in the pilot study, it was proposed to use the SNAS score to reflect the overall appearance.

However, in the current study, only a moderate correlation was found and therefore we recommend using the SNAS for assessment of symmetry solely. Hence, overall submental appearance (ie, aesthetics) could be considered as a different outcome.

The majority of methods using 2D quantitative media and 3D media to assess appearance after UCLP repair were described in the reviews of Al-Omari et al. (2005), Sharma et al. (2012), and Mosmuller et al. (2013). Although these methods often exhibit reliability values near to perfect, it can be concluded that the SNAS is far more easy-to-use and does not require expensive technical expertise. The lack of a simple and easy-to-use reliable assessment method to assess large number of patient’s photographs formed the main reason for creating the SNAS, since patient photographs are mostly readily available in almost every treatment center and can be used for relative quick comparison (Mosmuller et al., 2013). The reason for choosing the submental view was the ability of this view to expose several key anatomic structures (nostrils, columella, etc) that easily can be assessed according to symmetry. Other advantages can be mentioned concerning the SNAS: Only a single rater is required to obtain a reliability higher than 0.70. Again, with 3 raters the reliability will increase nearly toward 0.90. Prior to the beginning of the assessments, only 3 practice photographs were needed for the raters to become familiar with the SNAS to instantly obtain an interrater reliability of 0.73. Reliability could even increase when more practice photographs are scored before performing a rating task or when raters become more experienced using the SNAS. Moreover, grading a single photograph took only 13.4 seconds on average is this study. This means that using the SNAS, even with retaining the 10-second intervals between photographs, raters are able to assess approximately 20 photographs within 10 minutes.

### Strengths and Limitations of Study Design

In the pilot study, reliability was tested on 24 photographs that were uniformly adjusted according to image quality, skin color, and degree of scarring. The 50 photographs used in the current study did not receive any form of adjustments and were placed in horizontal plane by approximation on the PowerPoint slide, while in the pilot study, the computer program SymNose (Pigott and Pigott, 2010) was used for exact horizontal calibration. Still, adequate reliability was obtained for the SNAS in the current study. This is a major advantage when the instrument is used during multidisciplinary team meetings or for assessment of large caseloads, as none of these time-consuming procedures need to be undertaken before assessment.

A few limitations need to be addressed. Intrarater reliability was not calculated in this study, as we consider that the interrater reliability is more important than the intrarater reliability. Moreover, high intrarater reliability (0.84) was already found in the pilot study.

The main limitation of the current study was the order of performing the assessment. In the pilot study, the 3 raters graded the overall appearance before they graded the nasal features according to symmetry with the SNAS. In the current study, however, the raters started with grading symmetry using the SNAS and immediately after they graded the overall appearance. It is unclear to what extent the raters realize that symmetry and overall appearance are different constructs and thus to what extent the scores on SNAS and overall appearance influence each other.

A final practical limitation needs to be addressed again. Atypical outcomes such as a smaller nostril or an inverted nostril axis, both on the cleft side were not included in the reference photographs of the SNAS. Although such outliers are rarely seen, the SNAS is not able to provide assistance in grading these. For those cases, it is suggested to just grade symmetry based on the difference between the cleft and noncleft side.

The SNAS could be an instrument to quickly investigate differences between surgeons, techniques, and treatment centers. It could be used independently or in combination with existing rating scales using the frontal and/or lateral view. It would be an ideal instrument to function as a preselection tool given the fact that 3D systems are often still time-consuming, but highly reliable. After preselection with the SNAS, more specific 3D methods can be utilized to focus on specific differences. Further research might be undertaken, to use the SNAS in combination with other 2D assessment methods like the CARS (Mosmuller et al., 2017).

Comparison to patient satisfaction is also desirable, as in the end, patient satisfaction is considered as a highly important outcome in UCLP treatment (Wong Riff et al., 2018; Zeraatkar et al., 2019). Applicability of the SNAS on live patients and other age groups (18-22 years) should be investigated as well. To what extent additions should be made to the SNAS to cover less frequently occurring outcomes, such as an inverted nostril axis (not included in the reference photographs) remains a topic for further discussion.

## Conclusion

The SNAS is a useful and reliable tool to assess nasal symmetry from the submental perspective in 6- to 9-year-old patients with repaired UCLP. Compared to the pilot study, similar interrater reliability was found, and again the SNAS was shown to be more reliable then the assessment of overall submental appearance. However, construct validity of the SNAS, as an instrument for overall appearance was not supported in the current study. Therefore, the SNAS should only be used to assess nasal symmetry.

## References

[bibr1-1055665620961968] Al-Omari I Millett DT Ayoub AF. Methods of assessment of cleft-related facial deformity: a review. Cleft Palate Craniofac J. 2005;42(2):145–156. 15748105 10.1597/02-149.1

[bibr2-1055665620961968] Asher-McDade C Roberts C Shaw WC Gallager C. Development of a method for rating nasolabial appearance in patients with clefts of the lip and palate. Cleft Palate Craniofac J. 1991;28(4):385–390. 1742308 10.1597/1545-1569_1991_028_0385_doamfr_2.3.co_2

[bibr3-1055665620961968] Baudouin JY Tiberghien G. Symmetry, averageness, and feature size in the facial attractiveness of women. Acta Psychol (Amster). 2004;117(3):313–332. 10.1016/j.actpsy.2004.07.002 15500809

[bibr4-1055665620961968] Bongaarts CA Prahl-Andersen B Bronkhorst EM Spauwen PH Mulder JW Vaandrager JM Kuijpers-Jagtman AM. Effect of infant orthopedics on facial appearance of toddlers with complete unilateral cleft lip and palate (Dutchcleft). Cleft Palate Craniofac J. 2008;45(4):407–413. 18616367 10.1597/07-043.1

[bibr5-1055665620961968] De Vet HCW Terwee CB Mokkink LB Knol DL. Measurement in Medicine: A Practical Guide. Cambridge University Press; 2011;5:96–149.

[bibr6-1055665620961968] He X Li H Shao Y Shi B. Objective measurements for grading the nasal esthetics on basal view in individuals with secondary cleft nasal deformity. Cleft Palate Craniofac J .2015;52(1):66–69. 24320822 10.1597/13-099

[bibr7-1055665620961968] He X Shi B Kamdar M Zheng Q Li S Wang Y. Development of a method for rating nasal appearance after cleft lip repair. J Plast Reconstr Aesthet Surg. 2009;62(11):1437–1441. 18789881 10.1016/j.bjps.2008.05.018

[bibr8-1055665620961968] Hinkle DE Wiersma W Jurs SG. Applied Statistics for the Behavioral Sciences. Houghton Mifflin; 2003.

[bibr9-1055665620961968] Knight ZL Ganske IM Deutsch CK Mulliken JB. The changing nasolabial dimensions following repair of unilateral cleft lip: an anthropometric study in late childhood. Plast Reconstr Surg. 2016;138(5):879–886. 27783002 10.1097/PRS.0000000000002655

[bibr10-1055665620961968] Mani M Reiser E Andlin-Sobocki A Skoog V Holmström M. Factors related to quality of life and satisfaction with nasal appearance in patients treated for unilateral cleft lip and palate. Cleft Palate Craniofac J. 2013;50(4):432–439. 22035039 10.1597/11-035

[bibr11-1055665620961968] Mosmuller DG Don Griot JP Bijnen CL Niessen FB. Scoring systems of cleft-related facial deformities: a review of literature. Cleft Palate Craniofac J. 2013;50(3):286–296. 23030761 10.1597/11-207

[bibr12-1055665620961968] Mosmuller DG Mennes LM Prahl C Kramer GJ Disse MA Van Couwelaar GM Frank BN Don Griot JP. The development of the cleft aesthetic rating scale: a new rating scale for the assessment of nasolabial appearance in complete unilateral cleft lip and palate patients. Cleft Palate Craniofac J. 2017;54(5):555–561. 27537493 10.1597/15-274

[bibr13-1055665620961968] Mulliken JB LaBrie RA. Fourth-dimensional changes in nasolabial dimensions following rotation-advancement repair of unilateral cleft lip. Plast Reconstr Surg. 2012;129(2):491–498. 22286429 10.1097/PRS.0b013e31822b69b4

[bibr14-1055665620961968] Pigott RW Pigott BB. Quantitative measurement of symmetry from photographs following surgery for unilateral cleft lip and palate. Cleft Palate Craniofac J. 2010;47(4):363–367. 19860513 10.1597/08-175.1

[bibr15-1055665620961968] Prahl C Prahl-Andersen B Van’t Hof MA Kuijpers-Jagtman AM. Infant orthopedics and facial appearance: a randomized clinical trial (dutchcleft). Cleft Palate Craniofac J. 2006;43(6):659–664. 17105328 10.1597/05-139

[bibr16-1055665620961968] Rubin MS Lowe KM Clouston S Shetye PR Warren SM Grayson BH. Basal view reference photographs for nasolabial appearance rating in unilateral cleft lip and palate. J Craniofac Surg. 2015;26(5):1548–1550. 26163840 10.1097/SCS.0000000000001846

[bibr17-1055665620961968] Sharma VP Bella H Cadier MM Pigott RW Goodacre TE Richard BM. Outcomes in facial aesthetics in cleft lip and palate surgery: a systematic review. J Plast Reconstr Aesthet Surg. 2012;65(9):1233–1245. 22591614 10.1016/j.bjps.2012.04.001

[bibr18-1055665620961968] Tan RA Isaac KV Ganske IM Mosmuller DG De Vet HC Don Griot JP Mulliken JB. Development of the Submental Nasal Appearance Scale for the assessment of repaired unilateral complete cleft lip: a pilot study. Cleft Palate Craniofac J. 2019;56(6):791–798 30463427 10.1177/1055665618811507 PMC6604250

[bibr19-1055665620961968] Wong Riff KW Tsangaris E Goodacre TE Forrest CR Lawson J Pusic AL Klassen AF. What matters to patients with cleft lip and/or palate: an international qualitative study informing the development of the CLEFT-Q. Cleft Palate Craniofac J. 2018;55(3):442–450. 29437508 10.1177/1055665617732854

[bibr20-1055665620961968] Zeraatkar M Ajami S Nadjmi N Faghihi SA Golkari A. A qualitative study of children’s quality of life in the context of living with cleft lip and palate. Pediatric Health Med Ther. 2019;10:13–20. 30697094 10.2147/PHMT.S173070 PMC6342148

